# Experiences of Dutch general practitioners and district nurses with involving care services and facilities in palliative care: a mixed methods study

**DOI:** 10.1186/s12913-018-3644-2

**Published:** 2018-11-08

**Authors:** Ian Koper, H. Roeline W. Pasman, Bregje D. Onwuteaka-Philipsen

**Affiliations:** Department of Public and Occupational Health, Amsterdam Public Health research institute, Amsterdam UMC, Vrije Universiteit Amsterdam, Van der Boechorststraat 7, NL-1081 BT Amsterdam, The Netherlands

**Keywords:** Palliative care, Primary care, Utilization of services

## Abstract

**Background:**

Generals practitioners (GPs) and district nurses (DNs) play a leading role in providing palliative care at home. Many services and facilities are available to support them in providing this complex care. This study aimed to examine the extent to which GPs and DNs involve these services, what their experiences are, and how involvement of these services and facilities can be improved.

**Methods:**

Sequential mixed methods consisting of an online questionnaire with structured and open questions completed by 108 GPs and 258 DNs, followed by three homogenous online focus groups with 8 GPs and 19 DNs, analyzed through open coding.

**Results:**

Most GPs reported that they sometimes or often involved palliative home care teams (99%), hospices (94%), and palliative care consultation services (93%). Most DNs reported sometimes or often involving volunteers (90%), hospices (88%), and spiritual caregivers (80%). The least involved services and facilities were psychologists and psychiatrists (51% and 50%) and social welfare (44% and 57%). Main reason for not involving services and facilities was ‘not needing’ them. If they had used them, most GPs and DNs (68–93%) reported solely positive experiences. Hardly anyone (0–3%) reported solely negative experiences with any of the services and the facilities. GPs and DNs suggested improvements in three areas: (1) establishment of local centers giving information on available services and facilities, (2) presentation of services and facilities in local multidisciplinary meetings, and (3) support organizations to proactively offer their facilities and services.

**Conclusion:**

Psychological, social, and spiritual services are involved less often, suggesting that the classic care model, which focuses strongly on somatic issues, is still well entrenched. More familiarity with services that can provide additional care in these areas, regarding their availability and their added value, could improve the quality of life for patients and relatives at the end of life.

## Background

Palliative care is complex care, addressing physical, psychosocial, and spiritual problems at the end of life [1]. When faced with a life-threatening illness, most people in the Netherlands prefer to die at home [2]. Dutch national policy states that palliative care should principally be provided in the primary care setting [3], where primary care professionals such as general practitioners (GPs, in some countries better known as family physicians) and district nurses (DNs, in some countries better known as community nurses) play a leading role in the care for patients with a life-threatening illness in the primary care setting [4–7]. In this coordinated care model [8], the primary care physician provide general palliative care and can refer to specialist palliative care in case of complex problems. Similar to other countries like the UK, Australia and Canada [9], general practitioners serve as gatekeepers to specialist care services.

In practice, meeting the multidimensional needs of patients and their relatives has proven to be difficult [10–12]. There are many services and facilities available that GPs and DNs can involve or refer to when providing palliative care in the primary care setting. While most studies on palliative care services focus on hospices, palliative care consultation services, and/or palliative home care teams [13–18], there are also studies showing the added value of involving other services in palliative care such as psychologists, volunteers, and spiritual caregivers [19–21]. Little is known about how often these services are involved in palliative care by GPs and DNs in the Netherlands, and what the experiences are of GPs and DNs when using these services and facilities.

The first aim of this study was therefore to investigate how often GPs and DNs involve healthcare services and facilities in palliative care and what their experiences are with these services and facilities. The second aim was to investigate what reasons GPs and DNs have for not involving these services and facilities. Finally, we wanted to investigate how GPs and DNs think palliative care support by services and facilities can be improved, and how these improvements can be achieved.

## Methods

### Design

This mixed methods study had a sequential exploratory design [22], consisting of two parts. The first part was an online questionnaire, investigating GPs’ and DNs’ use of and experiences with services and facilities in palliative care and their reasons for not involving them, which was available online from April 5, 2016 until August 5, 2016. The second part of the study consisted of homogenous online focus groups in which the insights from the online questionnaire were explored more in-depth, with a focus on how palliative care support by services and facilities can be improved, and improvements in palliative care can be achieved. All focus groups were held within a three-month time frame: the first focus group started September 26, 2016 and the final focus groups finished December 6, 2016. In this study, palliative care was described as ‘*care for people with a life-threatening illness or age-related decline in the final phase of their life.’* The Medical Ethics Committee of the VUmc approved this study beforehand (METc VUmc 2016.320).

### Participants

Potential participants were invited to participate by professional organizations, the national organization of palliative care networks (Fibula) and regional care support networks (ROS) through newsletters and websites. Participating organizations were the Dutch College of General Practitioners (NHG), the Advisory Board of General Practitioners on palliative care (PalHag) and the Dutch Nurses’ Association (V&VN). The inclusion criteria were: 1) working as a GP or DN in patient care, 2) having experience with palliative care, and 3) working in the Netherlands. Details of 108 GPs and 258 DNs from all over the Netherlands who participated in the online survey responded to the questions on services and facilities are shown in Table 1.

**Table 1:** Characteristics of participants in the online survey and online focus groups

	Online survey		Online focus groups		
	GP	DN	GP	DN group 1	DN group 2
	*n* = 108	*n* = 258	*n* = 8	*n* = 9	n = 10
Age (mean (range))	49 (30–64)	46 (21–66)	51 (39–59)	42 (33–52)	50 (35–57)
Gender (female)	71%	94%	4	9	8
Mean working hours per week	33	26	35	33	26
Working experience (mean years (range))	17 (1–38)	13 (1–42)	18 (6–30)	10 (1–20)	17 (3–34)
Having received any training in palliative care	50%	59%	6	5	10

In the final question of the online questionnaire, participants were asked if they were interested in participating in a focus group aimed at further investigating points for improvement in palliative care. Participants who expressed an interest were invited to participate in an e-mail containing information on the procedure, discussion topics, and the ground rules. Twenty-two GPs were invited, 11 responded, 10 agreed to participate and 8 actually did so in practice. The equivalent figures for DNs were 24, 24, 20 and 19. Their details are shown in Table 1. Although the recruitment strategy does not allow for response rates to be calculated, characteristics of the sample can be compared to the national population. Nationwide, GPs are 48 years old on average, working 30 h per week [23] while DNs are 45 years old and working 15 h per week on average [24]. Respondents in our sample were of similar age, while working slightly more hours per week. Comparing the gender distribution of the respondents to national figures (nationwide, 51% of GPs and 92% of DNs are women), the proportion of female GPs in our sample is rather high.

### Questionnaire

The questionnaire contained both open and structured questions. Participants were asked to say for nine specific services and facilities whether or not they involved these when providing palliative care. The services and facilities concerned were: hospices, palliative care consultation services, clinical pain specialists, palliative home care teams, case managers for palliative care/dementia, spiritual caregivers, psychologists/psychiatrists, social welfare, and volunteers in palliative care. The possible answers were: ‘*yes, often’, ‘yes, sometimes’*, and *‘no’*. If participants reported using a particular service or facility sometimes or often, they were subsequently asked to rate their experiences. The possible answers were: *‘solely positive experiences’, ‘mixed experiences’*, and ‘*solely negative experiences’*. If participants reported that they did not involve certain services or facilities, they were asked to indicate why they did not. The possible answers were that these service were *‘unavailable’* or *‘not needed’* (from their perspective), that the participants had *‘bad experiences’* in the past, perceived involving them as *‘not my task’*, or *‘other reason’.* Participants who choose ‘*other reason’* were asked to elaborate. Next, participants were asked in an open question how palliative care with regard to services and facilities could be improved.

### Online focus groups

The focus group discussions were held online [25], on a website with an interface similar to an online chat room. Participants logged into the website using an account name (their code name) and password provided by a moderator (IK). There they could respond under their code name to the questions posed by the moderator and to other respondents’ comments. One question was posed each working day at 10.00 am, except on Wednesdays. The moderator sent an e-mail to all participants notifying them when a new question was available. This e-mail contained a link to the website as well as an encouragement to read and respond to earlier questions and comments by other respondents. The website was accessible 24 h a day, from the moment the first question was presented until one week after the last question was presented. The participants could click on a question to read the question with its context, read earlier comments from other respondents and react to both the question and the earlier comments. If necessary, the moderator redirected the discussion with follow-up questions at any time. For instance, the moderator summarized previous comments and asked the participants to respond to the summary. Any personal information or information that identified specific individuals or organizations was depersonalized by the moderator. In order to explore the insights from the questionnaire related to services and facilities in more depth, two of the questions for the online focus groups were on this topic: 1) *how can the accessibility of services and facilities such as hospices, spiritual caregivers, and volunteers be improved?* and 2) *how can the availability of services and facilities be made more widely known to healthcare providers as well as patients and relatives?*

### Data analysis

Data from the structured questions was analyzed using IBM SPSS Statistics software (Version 20.0). Descriptives were used to analyze the participant characteristics, involvement of services and facilities, and the respondents’ experiences and reasons for not involving them. Differences between GPs and DNs in their involvement of services and facilities were tested for statistical significance using Fisher’s Exact Test.

Data from the open question on improvements regarding services and facilities and the online focus groups was analyzed (separately) using open coding [26]. The codes were derived from the data rather than being determined beforehand. IK analyzed and coded the data, after which the codes were checked by RP and discussed with RP and BO. During this process, codes underwent content and definition changes as the analysis progressed and relations between codes became apparent. We coded thirteen subcategories that could be grouped into three overarching categories: 1) availability of services and facilities, 2) referrals to services and facilities, and 3) other improvements.

## Results

### Involvement of services and facilities in palliative care

The services and facilities that most GPs used sometimes or often were palliative home care teams (99%), palliative care consultation services (95%), and hospices (94%). DNs most frequently mentioned involving volunteers (90%), hospices (90%), and spiritual caregivers (80%) sometimes or often. Furthermore, 75% of the GPs and 61% of the DNs said that they sometimes or often involved a pain specialist, 69% and 53% a case manager, 51% and 50% a psychologist or psychiatrist and 44% and 57% social welfare. All differences between GPs and DNs were statistically significant, except for the differences in involving hospices and psychologists or psychiatrists. An overview is shown in Table 2.

**Table 2:** Extent to which GPs and DNs involve services and facilities when providing palliative care (% sometimes or often)

	GP^a^ *N* = 108	DN^a^ *N* = 258
	%	%
Palliative home care team*	99	67
Palliative care consultation services*	95	74
Hospice	94	90
Volunteers in palliative care*	82	90
Clinical pain specialist*	75	61
Case manager for palliative care/dementia*	69	54
Psychologist/psychiatrist	51	50
Spiritual caregiver*	50	80
Social welfare*	44	57

^a^Less than 5% missing for all rows

*Statistically significant difference between GPs and DNs (*p* < 0.05)

### Experiences with services and facilities and reasons for not involving them

The majority of the GPs and DNs who used services and facilities in palliative care reported solely positive experiences with these services and facilities, with percentages ranging from 91% of GPs and 93% of DNs for hospices, palliative care consultation services, and palliative home care teams to 68% and 74% for pain specialists. The percentage of participants reporting mixed experiences – i.e. both positive and negative – with services and facilities ranged from 6 and 9% for palliative consultation services to 32% and 25% respectively for clinical pain specialists. Hardly anyone (0–3%) reported solely negative experiences with any of the services and the facilities. An overview is shown in Table 3.

**Table 3:** Experiences of GPs and DNs district nurses with services and facilities in palliative care (row %)

	General practitioners^a^				District nurses^a^			
	N	Solely positive %	Mixed %	Solely negative %	N	Solely positive %	Mixed %	Solely negative %
Palliative home care team	105	92	8	.	146	91	9	.
Palliative care consultation services	98	93	6	1	168	91	9	.
Hospice	101	92	8	.	217	93	7	.
Volunteers in palliative care	79	80	20	.	216	87	12	1
Clinical pain specialist	74	68	32	.	143	74	25	1
Case manager for palliative care	70	70	27	3	122	81	19	.
Psychologist/psychiatrist	51	82	16	2	115	71	27	2
Spiritual caregiver	53	83	17	.	193	87	12	1
Social welfare	43	74	26	.	126	75	25	.

^a^Less than 5% missing for all rows

GPs and DNs who reported not involving certain services and facilities were asked to indicate why they did not. For most services and facilities, GPs and DNs mentioned ‘not needed’ as the main reason not to involve those services and facilities. The exceptions for GPs concerned palliative care/dementia case managers (‘unavailable’), spiritual caregivers (‘not my job’), and volunteers in palliative care (‘don’t know them/where to find them’). For DNs the only exception concerned clinical pain specialists (‘not my job’). Services and facilities being unavailable, not knowing them or how to find them, or not considering it their job were mentioned less often as reasons for not involving those services and facilities. Having bad experiences with services and facilities in the past was rarely given as a reason not to involve them. A detailed overview can be found in Table 4.

**Table 4:** GPs’ and DNs’ reasons for not involving services and facilities (absolute numbers and %*)

Service/facility^a^	N	Not needed	Not my job	Don’t know them/where to find them	Unavailable	Bad experiences	Other/no reason specified
Clinical pain specialist							
GP	27	**15**	0	1	0	6	5
DN	93	37 (40%)	**44 (47%)**	6 (6%)	2	0	4
Case manager for palliative care							
GP	34	8	0	5	**16**	1	4
DN	116	**56 (48%)**	12 (10%)	10 (9%)	18 (16%)	1	19 (16%)
Spiritual caregiver							
GP	54	16 (30%)	**23 (43%)**	9 (17%)	4 (7%)	0	2
DN	51	**23 (45%)**	7 (14%)	3 (6%)	3 (6%)	1	14 (27%)
Psychologist/psychiatrist							
GP	52	**39 (75%)**	0	1	2	1	9 (17%)
DN	123	**57 (46%)**	35 (28%)	4	2	1	24 (20%)
Social welfare							
GP	61	**40 (66%)**	0	2	1	4 (7%)	14 (23%)
DN	103	**62 (60%)**	15 (15%)	1	3	1	21 (20%)

*Percentage shown if total *N* > 50; boldfaced values show the most often mentioned reason per service for both professions

^a^We excluded palliative home care teams, palliative care consultation services, hospices, and volunteers in palliative care as the vast majority of GPs and DNs said that they used these services and facilities sometimes or often

### Improving the involvement of services and facilities in palliative care

We asked the participants how palliative care with regard to services and facilities could be improved, and 144 participants (104 DNs and 40 GPs) mentioned one or more areas of improvement, which can be clustered in three different categories. Improvement in the availability of services and facilities was mentioned by 84 respondents. These included comments on the availability and capacity of hospices, and the availability and faster provision of tools (e.g. morphine pumps and adjustable beds) and medication. Improvements in referrals to services and facilities were mentioned by 29 respondents, commenting that spiritual caregivers, volunteers, and respite care should be called in more often. Other improvements, such as better information about and improved funding for the available services and facilities was also mentioned by 31 respondents. Quotes illustrating the suggestions can be found in Table 5. Sixty-three participants (24 GPs and 39 DNs) indicated that no improvements were necessary in the services and facilities in their area, or had no ideas for improvements.

**Table 5:** Quotes illustrating three categories of areas of improvement

Availability of services and facilities	
*“Intensive home care in particular in the final phase, which often comes unexpectedly, is not always available, because the [health insurance (ed.)] allowance is running out and some things can’t always be predicted.” (GP650)*	
*“There is no hospice [in our area]. A respite care facility is coming, possibly with palliative beds. A palliative ward in a nursing home has recently been opened and modernized: I don’t know the details.” (GP619)*	
Referrals to services and facilities	
*“In institutions, spiritual caregivers are available. In the home setting people have to arrange this themselves, sometimes in emergency situations, as well as tussle with the health insurers. And where can they find someone that they get on with as well? Obviously this rarely happens, even though I see a great need. Professional caregivers can deal with this to some extent, but they are restricted in their possibilities. As people increasingly want to die at home, I feel that every healthcare supplier should offer a spiritual caregiver or counsellor.”(DN 318)*	
*“With long-term palliative care recipients, there is a need for options for structural night care, for instance twice a week, so the relatives can get a proper night’s sleep.” (DN277)*	
Other improvements	
*“It would be nice if there was a write-up of where people are best off ordering things, it would be nice if we had a brochure that we could hand out.” (DN 257)*	
*“Involving a palliative care nursing specialist in our area is not always possible. Sometimes because the insurance company doesn’t have a contract with our organization, sometimes because another healthcare provider organization doesn’t have a nursing specialist but can’t or doesn’t want to involve me. Ascitic drainage at home still isn’t funded properly and there is no regional coverage, so it is not available to all patients.” (DN 820)*	

Nineteen DNs and eight GPs participated in three homogenous online focus groups, where we asked how improvements in the availability of services and facilities could be achieved. Analysis of the focus group data revealed two key ways to achieve this. First, a central point of contact was suggested that can provide healthcare providers as well as patients and relatives with information on the available services and facilities. This point of contact could be a person (e.g. a district nurse) or a regional center, and should be connected to the regional palliative care network, ensuring familiarity with all local services and facilities. Healthcare providers caring for a patient with a life-threatening illness could then approach this point of contact to get in touch with the necessary services or facilities.

Second, it was suggested that services such as spiritual caregivers, volunteers in palliative care and social welfare should play a more active role in promoting themselves to improve the familiarity of GPs and DNs with these services and facilities. For example, services and facilities should be given the opportunity to introduce themselves and make their availability known in local multidisciplinary meetings or in locally organized training sessions.

## Discussion

Our results show that most GPs and DNs in our sample sometimes or often involve hospices, consultation services, palliative home care teams, and volunteers in palliative care. Fewer GPs and DNs involve psychologists or social welfare when providing palliative care. The majority of GPs and DNs reported mainly positive experiences with the services and facilities they used. ‘Not needing’ services or facilities in their perspective was often reported as a reason for not involving them.

According to GPs and DNs, there should be more referrals to services and facilities. The availability of services and facilities is also mentioned as a point of improvement. A central desk providing information on services and facilities in the area, and actively promoting services among GPs and DNs to increase their awareness and familiarity could reduce barriers to using these services and facilities in palliative care.

### Reflections on level of involvement of services and facilities in palliative care

We found that most services and facilities were involved sometimes or often by at least two thirds of GPs and DNs. These findings differ from studies on palliative care service use from the patient perspective. One study showed that 29% of patients in the Netherlands received specialist palliative care (i.e. involvement of hospices, consultation services or palliative home care teams) in the last three months of life [27]. Another study showed that in 27% of all cases of patients who had died a non-sudden death, one or more supportive caregivers (i.e. palliative care consultants, pain specialists, psychologists or spiritual caregivers) had been involved in the care in the last month of life [28]. It is therefore crucial to realize that even if all healthcare providers were to report ‘sometimes or often’ involving services or facilities, this would not mean that all patients receive this additional care. At the same time it is important to realize that not all available services or facilities have to be involved in every case [29]. According to the Dutch national palliative care policy, palliative care is supposed to be delivered by generalists, supported where necessary by healthcare providers with expertise in palliative care [3]. If the generalist can fulfill the needs of the patient on their own, or if a patient does not want additional healthcare providers to be involved, the involvement of additional services and facilities is unnecessary. Still, our finding that around half of the GPs never involves a psychologist, spiritual caregiver or social welfare is concerning.

### Reflections on reasons for not involving services and facilities

The main reason GPs give for not involving a clinical pain specialist is that it is ‘not needed’. As somatic issues like pain are traditionally the focus of treatment, and patients’ physical needs at the end of life are often met [10], this reason may be justified. As mentioned above, if generalists can meet the needs of their patient, the involvement of specialists may not be necessary. Furthermore, GPs have been shown to discuss difficulties in managing pain in their patients with palliative care consultation services [30].

The main reason GPs do not involve palliative care case managers is that they are unavailable. We know from research that while case managers are indeed not available in every part of the country, GPs and DNs are not always aware of their availability in regions where they *are* available [31, 32]. DNs mainly reported not needing a case manager as the reason for not involving them. This may be related to DNs (either formally or informally) taking on the role of case manager themselves [33]. While 80% of the DNs reported sometimes or often involving spiritual caregivers, only half of the GPs reported doing so. The main reason for GPs not referring to spiritual caregivers was ‘not my task’. Some GPs elaborated on this reason, commenting that they leave it up to the patient to seek spiritual care if they need it. However, lack of awareness and lack of physician referrals have been shown to be important barriers for patients in the use of palliative care services in general [34]. Furthermore, as spiritual support at the end of life is associated with better quality of life [35–37], and GPs struggle to provide spiritual care to patients for various reasons [38, 39], a more proactive approach from GPs may be appropriate here.

Regarding the decision not to involve a psychologist or psychiatrist, both GPs and DNs mainly reported that these services were not needed. Psychological issues such as anxiety, depression, and delirium are not uncommon in patients with a life-threatening illness and their relatives, and previous research has shown that the psychosocial needs of patients and relatives are often unmet [10, 40–42]. In some of these cases, the involvement of a psychologist or psychiatrist may prove valuable. GPs and DNs may be unaware of the potential value of involving psychologists in palliative care for patients and their relatives alike. The same might be the case for the potential value of involving social welfare [43], another service where both GPs and DNs gave ‘not needed’ as the main reason for not involving this service.

### Improving services and facilities in palliative care

GPs and DNs mentioned two major points for improvement: the availability of tools and services such as hospice beds, and more referrals to certain services and facilities. In the online focus groups, GPs and DNs discussed how these improvements can be achieved. The suggested solution has two elements. The first is the establishment of well-publicized central points of contact that can be approached by healthcare providers and patients alike to get information on the available services and facilities. This may indeed be a solution when unavailability or not knowing how to reach services and facilities is the main reason not to use them. This element alone, however, may not be enough. ‘Not needing’ services was a major reason for not involving them, and this reason may partly be related to a lack of awareness of the added value of these services. This emphasizes the need to educate healthcare providers on the availability and value of services and facilities. A way to achieve this was suggested in the online focus groups as the second element of the solution: enabling services and facilities to present themselves to groups of GPs and DNs in local multidisciplinary meetings. These proactive presentations may improve familiarity with those services and facilities, giving otherwise unaware GPs and DNs more insight into the added value of these services.

### Implications from an international perspective

The Netherlands is one of the few countries with generalist-plus-specialist palliative care [8], while in many countries such as the US, the UK, Canada, Australia, palliative care is a medical specialty [44]. Still, similar to the Netherlands, in most countries the majority of palliative care is provided by generalist practitioners [27, 45–48]. Earlier research showed that in Belgium, Italy and Spain specialist palliative care services such as palliative home care teams, volunteers, social workers and psychologists were involved in 39–47% of patients in the last three months of life [27]. In Australia around 30% of decedents had received palliative care services at home [49] and a recent study in Canada showed that 52% of decedents with cancer had received at least one home palliative care service [50]. It would be interesting to see if in these countries, including the Netherlands, increased awareness of healthcare providers on the availability and the added value of services and facilities in palliative care in other countries indeed improves patients’ access to these services, as the respondents in the online focus group suggest. This is important for the sustainability of palliative care for all people who need it [8].

### Strengths and limitations

A strength of this study lies in the mixed methods design, allowing us to elaborate on findings from the quantitative data in a qualitative way. Also, the accessibility of the online questionnaire enabled GPs and DNs from all over the country to participate, potentially providing a nationwide view on palliative care services. Still, it is possible that responders were GPs and DNs who are more interested and involved in palliative care. The finding that our sample on average worked more hours and contained a higher proportion of female GPs may be related to that [51]. This may have led to an overestimation of the involvement of services and facilities in palliative care. On the other hand, it would decrease the chance that respondents indicated a service was unavailable while such a service, unbeknownst to the respondent, was actually available. Yet, the reasons not to involve services or facilities are reported from the healthcare provider’s perspective. Thus, when they reported to not involve or make use of a service or facility because it is ‘unavailable’ or ‘not needed’, it is impossible for us to know if this is actually the case and not caused by unawareness of availability or added value.

## Conclusion

Services and facilities in palliative care can help meet the multidimensional needs of patients and relatives. Our finding that psychological, social, and spiritual services are involved less often suggests that the classic care model, with the primary focus on somatic issues, is still well entrenched. While involvement of all available services and facilities is certainly not always needed or desired by patients and relatives, it may be beneficial to involve these services more often. More familiarity with services that can provide additional healthcare in these areas, both with regard to availability and added value, could improve the quality of life for patients and relatives at the end of life.

## Abbreviations

DN: district nurse
GP: general practitioner
